# Early prediction of gestational diabetes mellitus using machine learning-integrated metabolomic and clinical features

**DOI:** 10.3389/fendo.2025.1687146

**Published:** 2025-11-13

**Authors:** Qun Ji, Lan Gao, Haiwei Liu, Xiaofang Chen, Boxia Fu, Yingbei Lin, Fei Wang

**Affiliations:** 1 Department of Endocrinology, Hainan General Hospital, Hainan Affiliated Hospital of Hainan Medical University, Haikou, Hainan, China; 2 Department of Medical Record Management, Hainan General Hospital, Hainan Affiliated Hospital of Hainan Medical University, Haikou, Hainan, China

**Keywords:** gestational diabetes mellitus, metabolites, machine learning, early prediction, metabolomic profiling

## Abstract

**Background:**

Gestational diabetes mellitus (GDM), a prevalent metabolic disorder associated with pregnancy, which often postpones intervention until after metabolic complications have developed. This study seeks to develop an integrated predictive model that combines first trimester metabolomic signatures with established clinical risk factors to enable the early detection of high-risk pregnancies prior to the onset of irreversible metabolic damages.

**Methods:**

A total of 89 pregnant women [45 with GDM, 44 with normal glucose tolerance (NGT)] was recruited at Hainan Provincial People’s Hospital. Serum and urine samples were subjected to untargeted metabolomic profiling employing UPLC-MS/MS. Metabolite identification was conducted using the Human Metabolome Database and Metlin databases. Bioinformatics analyses were performed on the differential metabolites. Lasso regression was employed to select the metabolites and clinical features utilized in constructing the model. The entire dataset was divided into a training set and a validation set in a 7:3 ratio. Six Machine learning models were trained to identify patients with GDM. Model performance was assessed using area under the receiver operating characteristic curve (AUC), precision, recall, and F1-score. Shapley Additive exPlanations (SHAP) analysis was used to interpret feature contributions in the optimal model.

**Results:**

Cases of GDM demonstrated distinct metabolic profiles in comparison to participants with NGT. A total of 528 differential metabolites were identified, and KEGG pathway analysis mapped these metabolites to 20 pathways related to metabolism and human diseases. Lasso regression identified 11 differential metabolites and 3 clinical features for training the ML models. Ultimately, the multilayer perceptron achieved the highest classification performance, with an AUC of 0.984 (95%CI: 0.866-1.000) in the validation set. SHAP analysis identified GlcCer(d18:1/16:0) and triglycerides as the most significant predictors, demonstrating positive associations with the risk of GDM.

**Conclusion:**

Participants with GDM and NGT show great difference in the levels of many metabolites. The ML model according to the metabolites in the first trimester and clinical feature demonstrates high accuracy for early GDM prediction. The result of this research highlighted the potential of metabolites in the prediction of GDM in the early stage of pregnancy.

## Introduction

Gestational diabetes mellitus (GDM), defined as glucose intolerance first identified during pregnancy that does not meet the criteria for overt diabetes mellitus which is one of the most common pregnancy-related complications (1). With changing of lifestyle and the rising age of pregnancy, the global prevalence of GDM has increased to approximately 14%, with rates reaching as high as 14.8% in China (2, 3). GDM not only increase risks of adverse pregnancy and perinatal outcomes but also exerts long-term health impacts on both mothers and offspring, demonstrating a “transgenerational effect” (4, 5). Notably, the incidence of GDM increased further during the COVID-19 pandemic, and infected GDM patients faced a 3.3-fold higher risk of intensive care unit admission compared to non-GDM pregnant individuals (6). Thus, early diagnosis and timely intervention for GDM are critical to reducing perinatal complications, mortality and safeguarding maternal and fetal health.

The current gold standard for GDM diagnosis is the oral glucose tolerance test (OGTT) performed at 24–28 weeks of gestation (7). However, OGTT is time-consuming, uncomfortable, requires specialized facilities and personal and delays diagnosis, prolonging fetal exposure to hyperglycemia - a limitation exacerbated during public health crises such as the COVID-19 pandemic (8). In response, simplified screening strategies, including glycated hemoglobin, fasting plasma glucose, and risk assessment models incorporating metabolic indicators, have been proposed globally whereas these approaches lack standardized criteria and sufficient specificity (9). Consequently, exploring simplified and efficient screening and diagnostic methods at early stage remains imperative for GDM management.

Metabolomics, a high-throughput analytical approach utilizing nuclear magnetic resonance spectroscopy or mass spectrometry, enables qualitative and quantitative detection of small-molecule metabolites and metabolic pathway analysis. Compared to genomics and proteomics, metabolomics offers closer detection to disease phenotypes, greater capacity to elucidate pathophysiological mechanisms and simpler analytical workflows (10). Metabolomic alterations usually occur in the early stage of disease progression which facilitating the identification of disease-specific biomarkers (10). This technique has proven valuable for early screening of complex, insidious conditions such as cancers, inflammatory bowel disease and diabetic nephropathy (11). Similarly, GDM is associated with distinct metabolomic changes. A study conducted by Leitner et al. identified significantly elevated levels of 2-hydroxybutyrate, tryptophan and serotonin in the blood and urine of GDM patients (12). A study conducted by Mokkala et al. which published in J Nutr demonstrated higher concentrations of two branched-chain amino acids and GlycA in overweight GDM patients during early pregnancy compared to non-GDM controls (13). A 2021 prospective cohort study from Central South University revealed 26 differential metabolites at 12–16 weeks of gestation associated with GDM onset and perinatal complications, suggesting their potential as early predictive markers (14, 15). These findings highlight the promise of metabolomics in advancing GDM diagnosis and treatment. However, GDM pathogenesis involves multifactorial mechanisms, and relying solely on metabolomic biomarkers while neglecting established clinical risk factors, such as advanced maternal age, obesity, multifetal gestation and family history of diabetes. The above factors are obviously not sufficient for an accurate diagnosis (16). In order to overcome the above limitations, a multi-dimensional prediction model based on the metabolomics characteristics of GDM patients combined with clinical characteristics is more suitable for practical clinical significance.

In clinical practice, rapid risk stratification for disease prediction is a common concern of healthcare providers and patients. Clinical prediction models which work as quantitative tools for risk-benefit assessment, providing more intuitive and rational information for doctors and patients to make decisions (17). Among these, machine learning plays a pivotal role in model construction. In contemporary medical research, machine learning (ML) algorithms have gained significant traction as analytical tools for deciphering complex biomedical datasets (18, 19). Accumulating evidence suggests these computational models demonstrate enhanced predictive capabilities compared to traditional statistical frameworks, particularly in scenarios involving intricate non-linear interactions among clinical variables (20). Despite these developments, the application of advanced ML methodologies, particularly ensemble learning approaches that combine multiple classifiers – remains underexplored in prognostic studies of thymic malignancies.

In summary, this study aims to establish a prospective cohort spanning early to mid-late stage of pregnancy, combining with metabolomics and ML to develop a clinical prediction model for GDM. By identifying differential metabolites between GDM and non-GDM pregnancies in early gestation and integrating these metabolomic signatures with clinical variables into a multidimensional model, we seek to enhance diagnostic accuracy and therapeutic outcomes. This approach will provide clinicians with an intuitive tool for early risk assessment, ultimately improving GDM diagnosis and maternal-fetal health outcomes.

## Methods

### Participants recruitment

A total of 89 participants were enrolled from January 1, 2023, to March 31, 2024, at Hainan Provincial People’s Hospital (Hainan, China). The participants were divided into two groups: participants with normal glucose tolerance (NGT, n=44) and the GDM patients’ group (n = 45). All patients were screened and diagnosed in accordance with the 2019 guidelines established by the American Diabetes Association. The diagnosis of GDM was determined based on the results of the 75g OGTT conducted between 24 and 28 weeks of gestation. Specifically, a diagnosis of GDM was made if any of the following criteria were met: a fasting blood glucose level of ≥5.1 mmol/L, a blood glucose level of ≥10.0 mmol/L one-hour post-glucose ingestion, or a blood glucose level of ≥8.5 mmol/L two hours post-glucose ingestion. Additionally, pregnant women who exhibited normal mid-pregnancy OGTT results but had a fasting blood glucose level of ≥5.1 mmol/L during subsequent pregnancy follow-up were also diagnosed with GDM. Those who did not meet these criteria were classified as NGT participants. Subjects with any of the following conditions were excluded: those with a previously established diagnosis of diabetes; pregnant women with twin or multiple pregnancies; those with a baseline fasting blood glucose ≥ 7.0 mmol/L, which meets the diabetes diagnostic criteria; those with severe acute or chronic diseases, such as severe liver function abnormalities (transaminases more than 3 times the upper limit of normal), renal function abnormalities (creatinine higher than the upper limit of normal), thyroid function abnormalities, cardiovascular and cerebrovascular diseases, respiratory diseases such as pneumonia and pulmonary tuberculosis, autoimmune diseases such as systemic lupus erythematosus and rheumatoid arthritis, hematological or tumor - related diseases, etc.; those with urinary system diseases, such as acute or chronic urinary tract infections, nephritis, cystitis, or unexplained hematuria and proteinuria; and those whose subjects themselves or their families were unable to understand and cooperate with this study. All procedures adhered to the principles of the Declaration of Helsinki and this research was approved by the ethics committee of Hainan Provincial People’s Hospital (No. Med-Eth-Re [2025] 574). Informed consent was obtained in writing from each participant upon their enrollment in the study.

### Metabolomics sample preparation and data acquisition

Samples from the GDM group and the NGT group were matched based on age, family history of diabetes, and pre - pregnancy body mass index. Peripheral blood and urine of the patients were used for metabolite extraction. Non-targeted metabolomics analysis was performed using an ultra - high performance liquid chromatography - tandem mass spectrometry (UPLC - MS/MS) system (ACQUITY UPLC-Xevo TQ-S, Waters Corporation, Milford, Massachusetts, USA). Quality control (QC) samples were obtained by regularly injecting a mixture of equal amounts of each sample. Raw data files were acquired using UPLC-MS/MS. Peak integration, calibration, and quantification of each metabolite were carried out using MassLynx software (v4.1, Waters, Milford, MA, USA). Metabolites with a relative standard deviation of > 30% in QC samples or present in less than 80% of the samples in any group were excluded from further analysis. Missing values were imputed with the minimum value, and the abundance data were log2-transformed.

### Metabolomics data processing

The raw mass spectrometry data were processed using Progenesis QI software. For the retrieval and identification of metabolites, the Human Metabolome Database (HMDB) and the Metlin database were used, and MetaAnalyst 5.0 was employed for data pre - processing. For the data exported from the QI software, variables with a coefficient of variance of less than 50% in the QC samples were screened. MetaboAnalyst 5.0 was used for data pre - processing, and the obtained data were used for qualitative analysis and multivariate statistical analysis. A correlation analysis was conducted on the QC samples to evaluate the stability of the instrument status during the experiment. The pre-processed data were imported into SIMCA software for analysis.

### Model construction

To mitigate overfitting, a generalized linear model incorporating a LASSO penalty was utilized to select features from metabolites that were significant according to the false discovery rate (FDR). The regularization parameter, λ, was determined through a 10-fold cross-validation process. The entire dataset was stratified into training set and validation set in a ratio of 7:3. Six ML models, including logic regression, decision tree, random forest (RF), extreme gradient boosting, support vector machine, multilayer perceptron (MLP) was trained with the data in training set. **Supplementary Table S1** provide a brief introduction of the above-mentioned models. Hyperparameters optimizing were conducted by grid search with 10-fold cross-validation process and the hyperparameters space can be found in **Supplementary Table S1**. Metrics including precision, recall, f1-score, accuracy and area under the receiver operating characteristic curve (AUC) and confusion matrix will be used in the assessment of models. Using bootstrapping with 1000 iterations, the 95% confidence intervals for the metrics were estimated.

### Model interpretation

To clarify the feature contributions of our models, Shapley Additive exPlanations (SHAP) library in the python will be used to calculate the shapley value of the best model.

### Statistical analysis

Continuous variables were expressed as means ± standard deviation (SD) and analyzed using the Student’s t-test when they followed a normal distribution. In cases where the data were not normally distributed, the variables were reported as medians with interquartile ranges and compared using the Wilcoxon rank-sum test. Categorical variables were expressed as frequencies and percentages, and comparisons were conducted using either the Chi-square test or Fisher’s exact test. Significant differences between metabolites were identified with an FDR below 0.05. The Benjamini–Hochberg method was used to correct for FDR in multiple metabolite comparisons. The inter-group differences in metabolites were comprehensively assessed and visualized using principal component analysis (PCA), partial least squares discriminant analysis (PLS-DA), and orthogonal partial least squares discriminant analysis (OPLS-DA) with R package ropls. The robustness of the model was evaluated through 7-fold cross-validation, and the permutation test (n = 1000 times) was used to verify the risk of overfitting. All statistical tests were two-tailed, with a P-value of less than 0.05 considered indicative of statistical significance. Statistical analyses were executed using R software, version 4.2.1 (R Project for Statistical Computing, Vienna, Austria).

## Results

### Demographic and clinical characteristics of participants

According to the inclusion and exclusion criteria, 89 participants were enrolled in this study and categorized into two groups: GDM patients and healthy individuals with NGT. The GDM group comprised 45 females with a median age of 32 years (range: 28–41 years), while the NGT group included 44 females with a median age of 32 years (range: 24–42 years). Detailed clinical characteristics are presented in **Table 1** and **Supplementary Table S2**. Notably, participants in the GDM group exhibited significantly higher body mass index levels compared to those in the NGT group (24.73± 4.05 vs. 21.81 ± 3.18, P < 0.001). This disparity might be associated with differences in physical activity duration, pre-pregnancy weight, and high-density lipoprotein cholesterol levels (all P < 0.05). No significant between-group differences were observed for most other clinical features (P > 0.05).

**Table 1:** Demographic and clinical characteristics of participants.

Variables	Overall (N=89)	GDM (n=45)	NGT (n=44)	P-value
Age (median [IQR])	32 [28, 35]	32 [28, 35]	32 [28, 34]	0.479
Body mass index (mean (SD))	23.29 (3.91)	24.73 (4.05)	21.81 (3.18)	<0.001
Educational level (%)				0.425
Middle school	15 (16.9)	28 (62.2)	30 (68.2)	
High school	15 (16.9)	10 (22.2)	5 (11.4)	
Bachelor	58 (65.2)	7 (15.6)	8 (18.2)	
Postgraduate	1 (1.1)	0 (0.0)	1 (2.3)	
Occupation (%)				0.965
Employee	28 (31.5)	15 (33.3)	13 (29.5)	
Public	22 (24.7)	11 (24.4)	11 (25.0)	
Self-employed	13 (14.6)	7 (15.6)	6 (13.6)	
Service	3 (3.4)	1 (2.2)	2 (4.5)	
Unemployed	23 (25.8)	11 (24.4)	12 (27.3)	
Physical labor time (%)				0.010
Greater than 30min	58 (65.2)	23 (51.1)	35 (79.5)	
Less than 30min	31 (34.8)	22 (48.9)	9 (20.5)	
Pregnancy planning (%)				0.347
Planed	68 (76.4)	32 (71.1)	36 (81.8)	
Unplaned	21 (23.6)	13 (28.9)	8 (18.2)	
Pre-pregnancy weight (median [IQR])	55.0 [50.5, 60.0]	57.0 [53.5, 65.0]	53.0 [46.9, 58.0]	0.002
Methods of conception (%)				0.751
Assisted	18 (20.2)	8 (17.8)	10 (22.7)	
Nature	71 (79.8)	37 (82.2)	34 (77.3)	
Gravidity (median [IQR])	2 [1, 3]	2 [2, 3]	2 [1, 3]	0.483
Parity (median [IQR])	0 [0, 1]	1 [0, 1]	0 [0, 1]	0.715
Abortion (median [IQR])	0 [0, 1]	0 [0, 1]	0 [0, 1]	0.975
Adverse pregnancy history (%)				0.163
No	49 (55.1)	21 (46.7)	28 (63.6)	
Yes	40 (44.9)	24 (53.3)	16 (36.4)	
Triglycerides (mean (SD))	1.94 (0.98)	2.12 (0.95)	1.67 (1.02)	<0.001
Total cholesterol (mean (SD))	5.33 (1.20)	5.28 (1.11)	5.38 (1.29)	0.699
High-density lipoprotein cholesterol (mean (SD))	2.05 (0.39)	1.95 (0.32)	2.14 (0.43)	0.016
Intracellular water content of left upper limb (mean (SD))	1.36 (0.30)	1.45 (0.31)	1.26 (0.26)	0.003
Fasting plasma insulin (mean (SD))	7.68 (1.32)	8.31 (1.15)	6.52 (1.24)	0.002
Low-density lipoprotein cholesterol (mean (SD))	6.03 (30.34)	9.18 (42.67)	2.82 (0.75)	0.325

IQR, interquartile range; SD, standard deviation.

### Comparison of serum metabolic profiles

**Figure 1:**
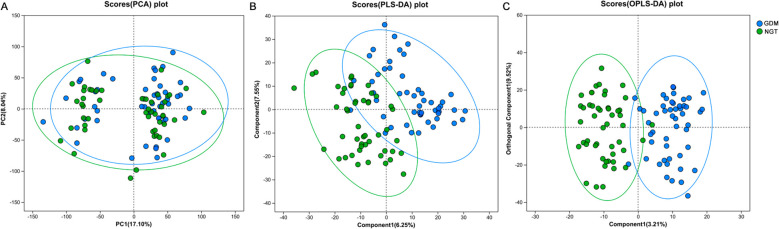
PCA scores, PLS-DA scores and OPLS-DA scores for the GDM group and the NGT group. **(A)** PCA score plot of the first two principal components (PC1 vs PC2). **(B)** PLS-DA score plot of the first two latent variables (component1 vs component2). **(C)** OPLS-DA score plot showing the predictive component versus the first orthogonal component. Each point represents one subject; group membership is indicated by color. Note: PCA, principal component analysis; PLS-DA, partial least squares discriminant analysis, OPLS-DA, orthogonal partial least squares discriminant analysis; GDM, patients with gestational diabetes mellitus; NGT, healthy individuals with normal glucose tolerance.

The PCA model was used to evaluate the serum metabolite profiles of the subjects. The results revealed different separation trends between the GDM and NGT groups (P = 0.027), indicating significant differences in their overall serum metabolic characteristics, as shown in **Figure 1A**. We further employed PLS-DA to conduct a more in-depth investigation of the serum metabolic characteristics of the two groups. This approach improved the discrimination between the groups and highlighted significant metabolic changes (P < 0.001), as illustrated in **Figure 1B**. Based on the PLS-DA model, an OPLS-DA model was further established for multi-dimensional analysis. **Figure 1C** shows an obvious separation trend between the GDM group and the NGT group (P < 0.001). All models showed a Q^2^Y value > 0.2 and a Y-axis intercept < 0 in the permutation test curve (**Supplementary Figure S1**), indicating non-overfitting and statistically significant differences in the metabolic profiles between groups. These findings reveal substantial metabolic differences between the GDM and NGT groups, thereby providing an essential basis for subsequent variable selection and the development of predictive models.

### Identification of differential metabolites between groups

**Figure 2:**
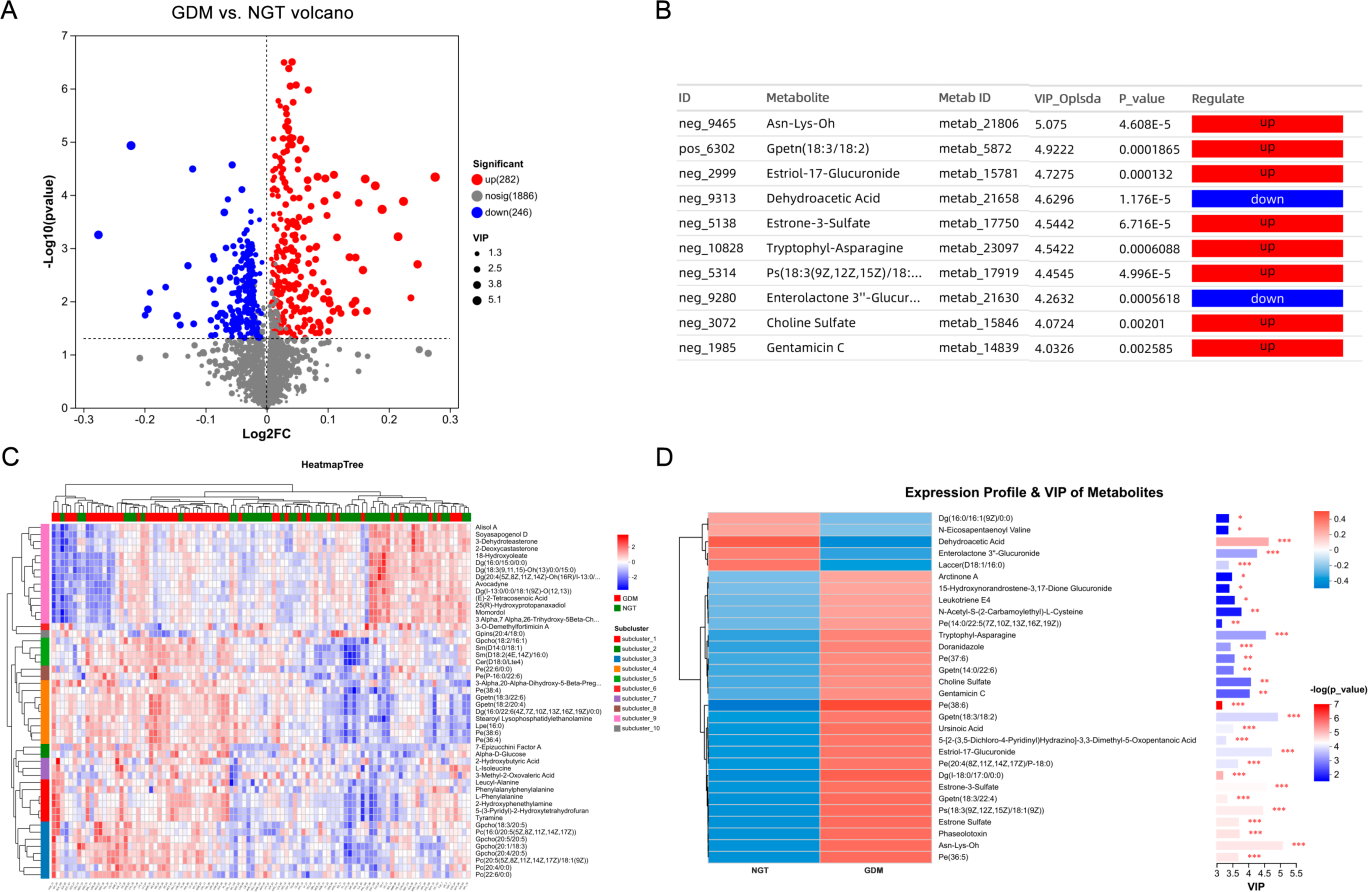
Metabolomics analysis of different groups. **(A)** This figure shows the differential expression of metabolites. Red dots represent the metabolites with upregulated expression in the GDM group (n = 275), and blue dots represent the metabolites with downregulated expression in the GDM group (n = 279). **(B)** The most important metabolites were identified via VIP. **(C)** The heatmap illustrates the expression of each metabolite in the two groups and the relationships among metabolites. **(D)** The heatmap of differential metabolites (P < 0.05, Student’s t test) represents the metabolite signal intensity of each sample, showing the relatively significantly increased and decreased metabolites in the two groups and their respective contribution degrees to the grouping model. Note: GDM, patients with Gestational Diabetes Mellitus; NGT, healthy individuals with Normal Glucose Tolerance; VIP: variable importance in the projection; *: P < 0.05; **: P < 0.01; ***: P < 0.001.

To accurately identify the LC-MS metabolites within the population affected by GDM, an OPLS-DA model was utilized, employing a variable importance in the projection (VIP) threshold greater than 1 and a significance level of P ≤ 0.05. This approach facilitated the identification of differential metabolites between the GDM group and the NGT group. A total of 528 metabolites were identified as potential biomarkers for GDM (**Figure 2A**). Within this set, 246 metabolites demonstrated a decrease, while 282 exhibited an increase in the GDM group. Notably, among the identified metabolites, Asn-Lys-Oh, Gpetn (18:3/18:2), and Estriol-17-Glucuronide were among the most significant (**Figure 2B**). Through dendrogram clustering analysis, the metabolites were clustered based on their correlations. Metabolites that were close to each other on the dendrogram had similar correlation patterns, suggesting that they might be associated in metabolic pathways or participate in similar physiological processes (**Figure 2C**). A detailed statistical analysis and hierarchical clustering of LC-MS data via MetaboAnalyst identified 528 metabolites with significant differences between the GDM and NGT groups. Notably, Pe (38:6) and Dg (I-18:0/17:0/0:0) were more abundant in the GDM group, whereas dehydroacetic acid levels were lower (**Figures 2C, D**).

**Figure 3:**
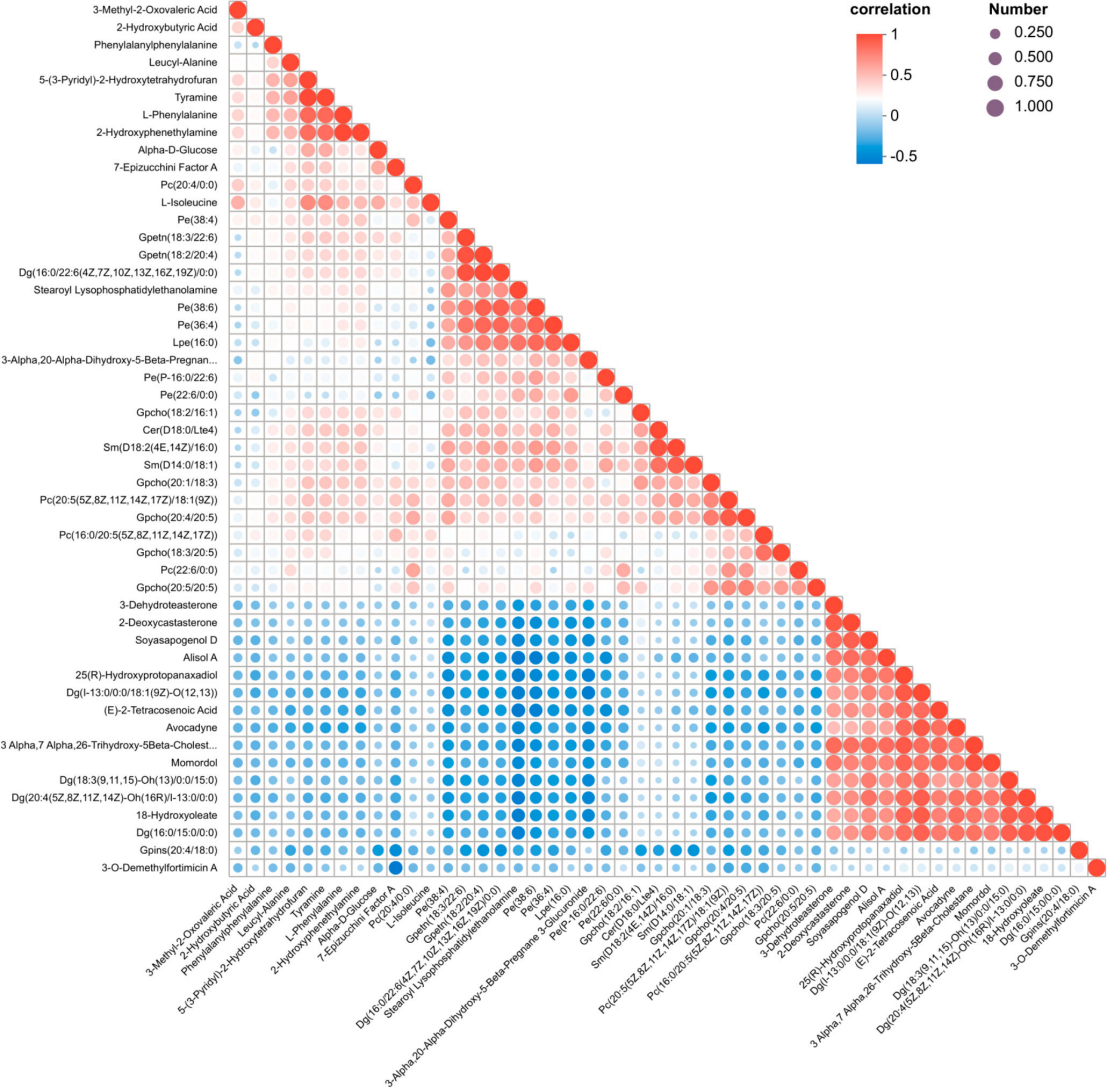
Correlations among differential metabolites.

We further conducted a correlation analysis on the 528 differential metabolites between the GDM group and the NGT group. Outside the diagonal line, significant correlations were found among some metabolites. For example, Tyramine showed a strong positive correlation with Leucylalanine, while 3-O-demethylfortimicin and 7-epizucchini. Factor A showed a strong negative correlation (**Figure 3**).

### KEGG enrichment analysis of differential metabolites

**Figure 4:**
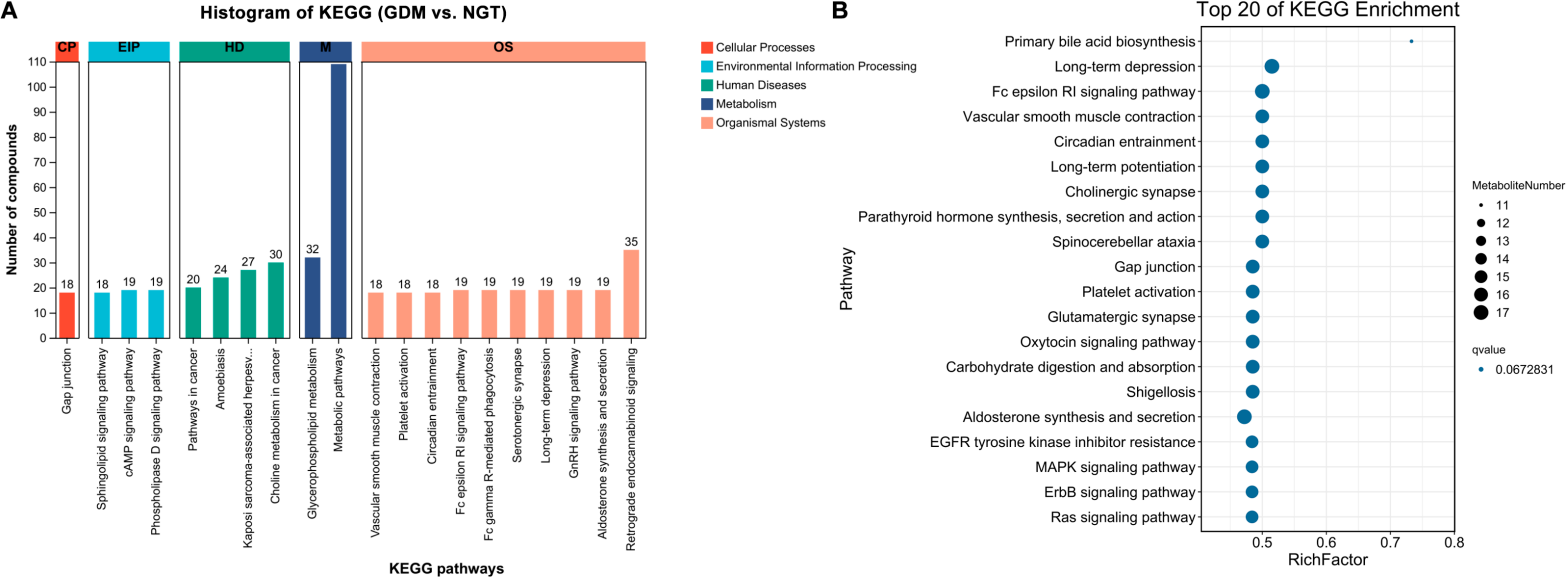
KEGG enrichment analysis. **(A)** KEGG enrichment analysis was performed on the differentially abundant metabolites in GDM and NGT based on the LC-MS data. According to the biological functions of metabolites, they are mainly classified into Metabolism, Environmental Information Processing, Cellular Processes, Organismal Systems, and Human Diseases. **(B)** The top 20 significantly enriched KEGG pathways are shown, presenting the biological pathways closely related to the metabolic phenotype of the GDM group. The size of the dots represents the number of metabolites enriched in this pathway. GDM, patients with Gestational Diabetes Mellitus; NGT, healthy individuals with Normal Glucose Tolerance.

We performed a pathway analysis on 528 differential metabolites between the GDM and NGT groups using MetaboAnalyst 5.0, generating pathway maps based on the KEGG database. The KEGG analysis of the 528 metabolites showed that 20 pathways were significantly enriched in GDM, which belonged to 5 categories, including metabolism, environmental information processing, cellular processes, organismal systems, and human diseases (**Figure 4**).

### Machine learning model construction and evaluation

**Figure 5:**
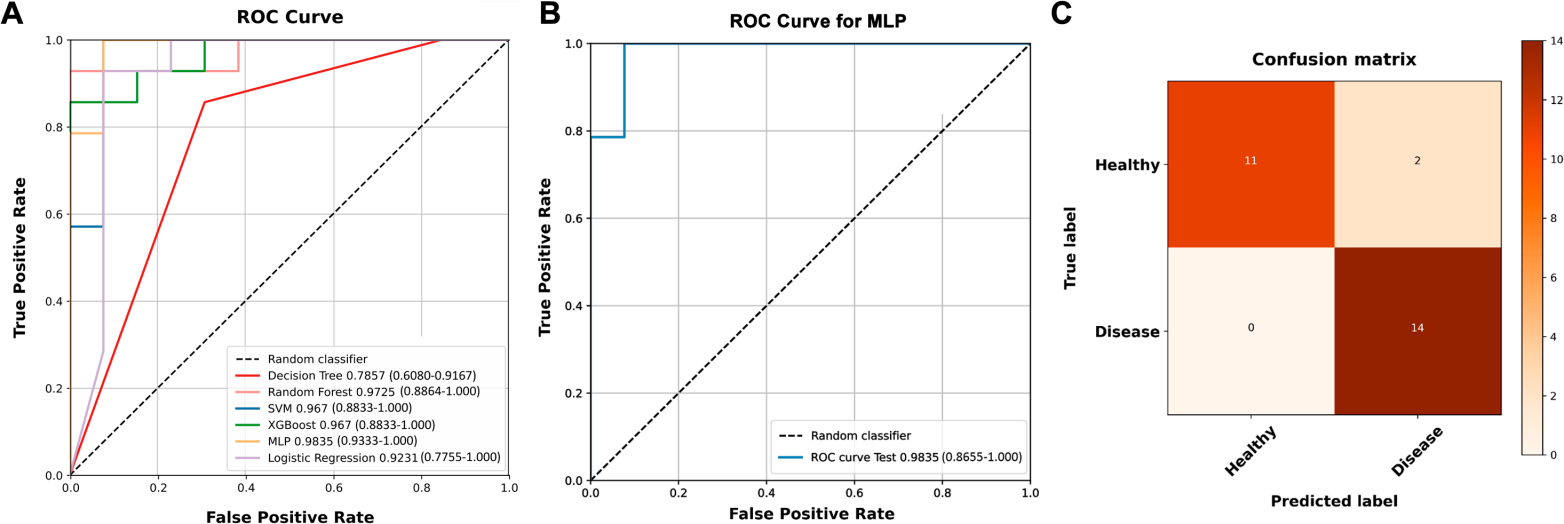
The performance of machine learning models. **(A)** Almost all the models have favorable performance in the validation set. **(B)** The MLP model performed best with highest AUC in both training set (AUC: 0.984, 95%CI: 0.933-1.000) and validation set (AUC: 0.984, 95%CI: 0.866-1.000). **(C)** Only two normal glucose tolerance sample were misclassified as patients with gestational diabetes mellitus by the MLP model. MLP: multilayer perceptron. AUC: area under receiver operating characteristic curve. CI: confidence intervals.

According to the result of LASSO regression, 14 metabolites were selected to construct the ML model. The information of these features can be found in **Supplementary Table S3**. All the presented models based on these features performed well in distinguishing patients with GDM (**Table 2**, **Figure 5A**). The best combinations of hyperparameters are presented in **Supplementary Table S4**. Among all the models, MLP performed best in both training set and validation set, with AUC of 0.984 (95%CI: 0.933-1.000) and 0.984 (95%CI: 0.866-1.000) respectively (Table 2, **Figure 5B**). According to the confusion matrix in the validation set, only 2 patients in the NGT group were misclassified into the GDM group, and all the GDM patients were distinguish by the MLP model (**Figure 5C**).

**Table 2:** Evaluation metrics of the models.

Models	Precision	Recall	F1-score	Accuracy
Decision tree	0.784 [0.625, 0.877]	0.775 [0.625, 0.877]	0.775 [0.667, 0.867]	0.778 [0.672, 0.853]
Random forest	0.892 [0.764, 0.959]	0.887 [0.739, 0.945]	0.888 [0.800, 0.952]	0.889 [0.800, 0.940]
XGBoost	0.892 [0.764, 0.959]	0.887 [0.739, 0.945]	0.888 [0.800, 0.952]	0.889 [0.800, 0.940]
SVM	0.912 [0.792, 0.973]	0.885 [0.739, 0.945]	0.886 [0.817, 0.961]	0.889 [0.815, 0.948]
MLP	0.938 [0.831, 0.986]	0.923 [0.801, 0.974]	0.925 [0.872, 0.987]	0.926 [0.862, 0.973]
LR	0.778 [0.654, 0.875]	1.000 [0.912, 1.000]	0.875 [0.800, 0.944]	0.852 [0.770, 0.921]

SVM, support vector machine; MLP, multilayer perceptron; LR, logistic regression.

### Feature explanation

**Figure 6:**
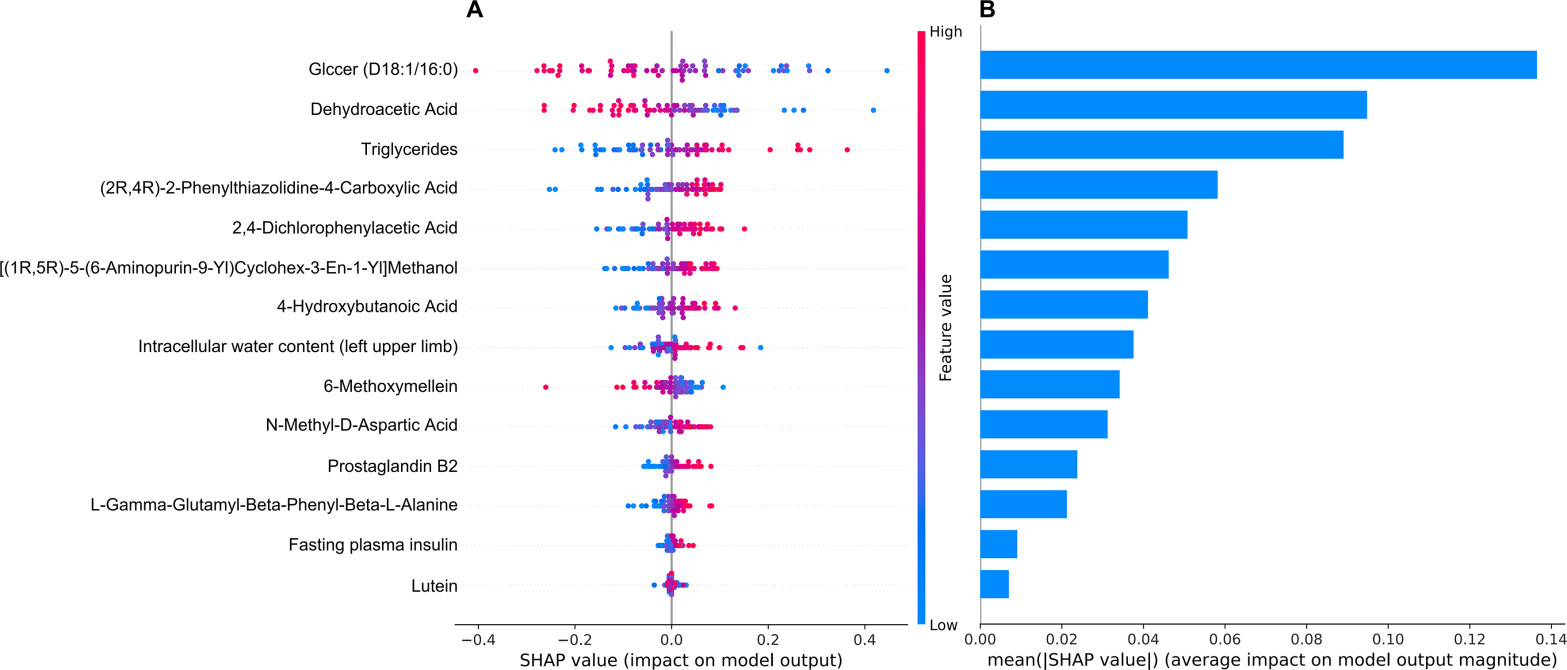
SHAP. The SHAP value of each sample and features were calculated **(A)**. The mean SHAP value of all samples for each variable will be used to rank the variable importance **(B)**. SHAP, Shapley Additive exPlanations.

We calculated the SHAP value of all the included features in all the samples with the best model MLP (**Figure 6A**). The mean absolute SHAP value of the features will be used to estimate the significance of each feature (**Figure 6B**). The magnitude of the SHAP value presented the degree of feature contribute to the prediction. The positive SHAP value indicated that the feature tends to increase the risk of GDM. Features including GlcCer(d18:1/16:0), dehydroacetic Acid, triglyceride, (2R,4R)-2-phenylthiazolidine-4-carboxylic acid and 2,4-dichlorophenylacetic acid are the top five features that contribute to the final decision of the MLP model.

## Discussion

With changes in lifestyle and the increase in the age of conception, the incidence of GDM is rising year by year, which is approximately 14% globally (2). GDM not only increases the risk of adverse outcomes during pregnancy and the perinatal period (5), but also affects the long-term health of both the mother and the child after childbirth. Therefore, early diagnosis and initiation of treatment for GDM are of great significance for reducing the risk of perinatal complications and mortality and ensuring the health of both the pregnant woman and the fetus. In this study, we apply metabolomics analysis on the peripheral blood samples of pregnant women between 10 and 20 weeks of gestation. Our results suggest that some of the metabolites such as GlcCer(d18:1/16:0) and dehydroacetic acid were different expressed in GDM patients and participants with NGT, and these metabolites may contribute to the prediction of GDM in the early stage of gestation. Based on these metabolites and the clinical indicators of the participants, we constructed seven models for the early detection of GDM patients. Ultimately, the MLP model demonstrated outstanding performance, achieving AUC values of 1.000 and 0.984 in the training and validation sets, respectively.

In general, all models involved in the study—including the less complex logistic regression—demonstrated strong performance in both the training and validation sets. This observation aligns with findings from similar studies (21). We speculate that this phenomenon may be attributed to the following reasons. Firstly, the variables incorporated into the ML analysis were carefully selected from an initial pool of 2,892 detected metabolites. After a series of screening steps, the final set of variables likely exhibits strong statistical and biologically plausible associations with the study outcome. Second, unlike most clinical studies, metabolomics research involves sample testing that requires prior planning. As a result, the case and control groups in this study were well-balanced in terms of sample size. Finally, all samples in this study were collected from a single center, and the validation set was derived from internal validation. This consistency across samples may have contributed to the apparently higher performance metrics of the models. This scenario evidently heightens the likelihood of model overfitting, particularly when the model is employed on datasets exhibiting substantially divergent feature distributions, potentially compromising its performance. Consequently, the findings of this study should be approached with careful consideration.

The relationship between metabolomics and GDM has been discussed in previous studies. Zhu et al. conducted untargeted metabolomic profiling of fasting serum samples from 91 GDM patients and 180 NGT participants at 10–13 and 16–19 weeks of gestation using gas chromatography/time-of-flight mass spectrometry (22). They found that purinone at 10–13 weeks was positively associated with GDM risk, while amino acids, amino alcohols, hexoses, indoles, and pyrimidine metabolites at 16–19 weeks were also positively correlated with GDM risk. Based on these metabolites, they developed multiple metabolite-based models for GDM prediction, achieving an AUC of 0.771–0.972 in the validation set. In 2023, Razo-Azamar et al. conducted a similar study using targeted metabolomics to analyze metabolite levels in 13 GDM patients and 62 NGT participants (21). By applying RF for metabolite selection and model construction, their final model achieved an AUC of 0.934. Lu et al. performed a large-scale metabolomic study involving 200 healthy pregnant women and 200 GDM patients during the second (24–27 weeks) and third trimesters (≥28 weeks). The most significantly differentially expressed metabolites were 3-methyl-2-oxovaleric acid in the second trimester and ketoleucine and alpha-ketoisovaleric acid in the third trimester (23). Their final models achieved AUCs of 0.807 and 0.810 for the second and third trimesters, respectively. Compared to previous studies, our research explores ML algorithms more extensively, not only training a wider variety of models and identifying one with superior performance (validation set AUC: 0.984) but also enhancing interpretability using SHAP. However, it is worth noting that sample size remains a common limitation in metabolomic studies, and further validation with larger cohorts is needed to confirm the robustness of our model.

In the realm of clinical practice, the early diagnosis and intervention of GDM are critically important due to its high prevalence and the substantial adverse effects on both maternal and fetal health (2, 3). Several studies have demonstrated promising outcomes utilizing machine learning models for the early prediction of GDM (22–25). Should these findings be effectively integrated into clinical practice, they hold the potential to markedly enhance healthcare outcomes. Nonetheless, it is imperative to address several significant challenges before these models can be comprehensively implemented in clinical environments. Firstly, the issue of model robustness presents a significant challenge. The majority of existing studies have been conducted within single-center, small-sample environments, thereby constraining the generalizability of the models. For these models to achieve broad clinical acceptance, they must demonstrate robustness across larger, multi-center studies, and their findings should be readily accessible to healthcare professionals (25, 26). Additionally, a critical challenge is the cost associated with metabolomic profiling. Despite technological advancements, the high expense of metabolic testing continues to impede its widespread clinical application. Future research should prioritize the development of cost-effective strategies for implementing metabolomic analysis or investigate alternative screening methods that can mitigate costs.

The objective of the metabolomics analysis in this study is to screen out the key differential metabolites between the groups of NGT and GDM. Differential metabolites such as GlcCer(D18:1/16:0), dehydroacetic acid, triglyceride, 2-phenylthiazolidine-4-carboxylic acid, 2,4-dichlorophenylacetic acid, [(1R,5R)-5-(6-aminopurin-9-yl)cyclohex-3-en-1-yl]methanol, 4-hydroxybutyric acid, 6-methoxymellein, N-methyl-D-aspartic acid, prostaglandin B2 (PGB2), L-γ-glutamyl-β-phenyl-β-L-alanine, and lutein are screened out. The strong predictive capabilities of the PLS-DA and OPLS-DA models prompted the link between these metabolites and sample groups, supporting further investigation into their role in GDM progression and offering potential early diagnosis biomarkers.

Dyslipidemia may be an important characteristic of GDM. GlcCer(D18:1/16:0) is a type of sphingolipid. Sphingolipids mainly exist in nerve tissues and are important components of the cell membranes of the brain and nerves. It plays critical roles in cellular membrane architecture, signal transduction, and the pathogenesis of various diseases, including diabetes mellitus. Although existing studies have not directly explored the relationship between GlcCer(D18:1/16:0) and GDM, previous studies have indicated that gangliosides GM1 and GM2 can effectively inhibit the phosphorylation of insulin receptors *in vitro (*27). Tagami et al. demonstrated that exogenous addition of GM3 to adipocytes can inhibit insulin receptor phosphorylation and affect glucose uptake (28). These findings indirectly suggest that there may be an association between sphingolipids and insulin function, which provides a reference for understanding the role of GlcCer (D18:1/16:0) in GDM.

Another commonly observed feature of GDM is insulin resistance. Studies have shown that patients with GDM often exhibit dyslipidemia, including elevated triglycerides (TG) and reduced high-density lipoprotein cholesterol, which could contribute to altered insulin sensitivity. These dyslipidemias may affect insulin sensitivity, thereby exacerbating the condition of GDM. During pregnancy, in order to ensure the nutritional supply of the fetus, the mother’s body will develop some insulin resistance. However, if the pregnant woman has problems such as obesity and hyperlipidemia, this physiological insulin resistance may be excessive, leading to the development of GDM. Hypertriglyceridemia is a common manifestation of dyslipidemia in diabetic patients. TG, serving as the principal constituents of lipids, play a pivotal role in energy metabolism and regulatory processes (29). Insulin resistance leads to impaired glucose utilization and simultaneously promotes lipolysis, increasing the release of free fatty acids, which further aggravates insulin resistance and forms a vicious cycle. Excessive free fatty acids are used to synthesize TG in the liver, resulting in an increase in blood triglyceride levels (30).

Pancreatic islet β-cells are central to maintaining blood glucose homeostasis, and their dysfunction and reduction in number have been identified as contributing factors in the progression to diabetes. Many substances have the potential to cause damage to islet β-cells and affect insulin secretion. Studies have shown that N-Methyl-D-Aspartate (NMDA) receptors play a role in islet β-cell function, vasodilation, neuroprotection, and diabetes-related complications. Through research on pancreatic BRIN-BD11 β-cells, Patterson S et al. found that NMDA receptors can regulate cellular insulin content and release, changes in membrane potential, intracellular Ca^2+^, and gene expression (31). Another study also indicated that NMDA may inhibit the expression of pancreatic ATP-binding cassette transporter A1(ABCA1) through the MEK/ERK/LXR pathway (32). ABCA1 plays a crucial role in the reverse cholesterol transport system, and its decreased expression is associated with lipotoxicity in pancreatic β-cells, leading to abnormal insulin synthesis and secretion. In addition, certain substances can affect insulin secretion through neuroendocrine effects.

This study also identified significant differences in 6-methoxymellein and L-gamma-glutamyl-beta-phenyl-beta-L-alanine between GDM and NGT finally, with no prior reports linking these metabolites to diabetes. In addition, although this study suggests that several other metabolites may be associated with the risk of GDM, relevant research remains scarce. Despite the lack of existing literature support, the differential expression of these metabolites may provide new insights into unaccounted metabolic abnormalities in GDM patients, potentially opening up new avenues for future diabetes research.

Nevertheless, several limitations warrant consideration. Firstly, the limited sample size and homogeneous characteristics of participants from Hainan Provincial People’s Hospital may substantially elevate the risk of overfitting and constrain the model’s generalizability. Future research should focus on increasing the sample size to enhance the model’s ability to learn features more effectively. Additionally, incorporating external datasets for further validation should be included to demonstrate the model’s robustness. Additionally, the small validation set (n≈27) may affect the robustness of the model evaluation, and the results should be interpreted with caution. Secondly, this study has only confirmed the differences in metabolites between the GDM group and the NGT group and has not conducted in-depth research on their specific mechanisms of action. Due to the lack of relevant literature support, the current speculations about some differential metabolites still require more experimental verification. Future research can be explored in depth through the following directions: First, use cell models to study the effects of metabolites on the function of islet β-cells and insulin sensitivity; Second, verify their role in the progression of diabetes through animal experiments; Finally, combine multi-omics technologies (such as transcriptomics and proteomics) to comprehensively reveal the metabolic pathways and regulatory networks in which metabolites are involved, and clarify their potential value as biomarkers or therapeutic targets for diabetes.

## Conclusion

This study found that the combination of metabolites and clinical indicators in early pregnancy holds significant importance in predicting the risk of GDM. The final MLP model trained in the study achieved an AUC of 0.984 in the validation set. Furthermore, the metabolites identified as key predictors potentially involve in lipid metabolism disorders, insulin resistance, and impaired β-cell function according to established pathophysiological mechanisms of GDM.

## Data Availability

The raw data supporting the conclusions of this article will be made available by the authors, without undue reservation.

## Supplementary material

Supplementary Figure 1 Permutation test for PLS-DA and OPLS-DA. **(A, B)** Permutation test for PLS-DA. **(C, D)** Permutation test for OPLS-DA. PLS-DA, partial least squares discriminant analysis, OPLS-DA, orthogonal partial least squares discriminant analysis.
