# Correction: Early prediction of gestational diabetes mellitus using machine learning-integrated metabolomic and clinical features

**DOI:** 10.3389/fendo.2026.1810108

**Published:** 2026-02-25

**Authors:** Qun Ji, Lan Gao, Haiwei Liu, Xiaofang Chen, Boxia Fu, Yingbei Lin, Fei Wang

**Affiliations:** 1 Department of Endocrinology, Hainan General Hospital, Hainan Affiliated Hospital of Hainan Medical University, Haikou, Hainan, China; 2 Department of Medical Record Management, Hainan General Hospital, Hainan Affiliated Hospital of Hainan Medical University, Haikou, Hainan, China

**Keywords:** gestational diabetes mellitus, metabolites, machine learning, early prediction, metabolomic profiling

Affiliation “^1^Department of Endocrinology, Hainan General Hospital, Hainan Affiliated Hospital of Hainan Medical University, Haikou, Hainan, China” was erroneously given as “^1^Department of Endocrinology, Hainan Provincial People’s Hospital, Hainan Affiliated Hospital of Hainan Medical University, Haikou, Hainan, China”. Affiliation “^2^Department of Medical Record Management, Hainan General Hospital, Hainan Affiliated Hospital of Hainan Medical University, Haikou, Hainan, China” was erroneously given as “^2^Department of Medical Record Management, Hainan Provincial People’s Hospital, Hainan Affiliated Hospital of Hainan Medical University, Haikou, Hainan, China”.

The original version of this article has been updated.

