# Supplementary figures and images for: Early prediction of gestational diabetes mellitus using machine learning-integrated metabolomic and clinical features
Source: Front Endocrinol (Lausanne). 2025 Nov 13;16:1687146. doi: 10.3389/fendo.2025.1687146 (PMC12658359; doi:10.3389/fendo.2025.1687146)

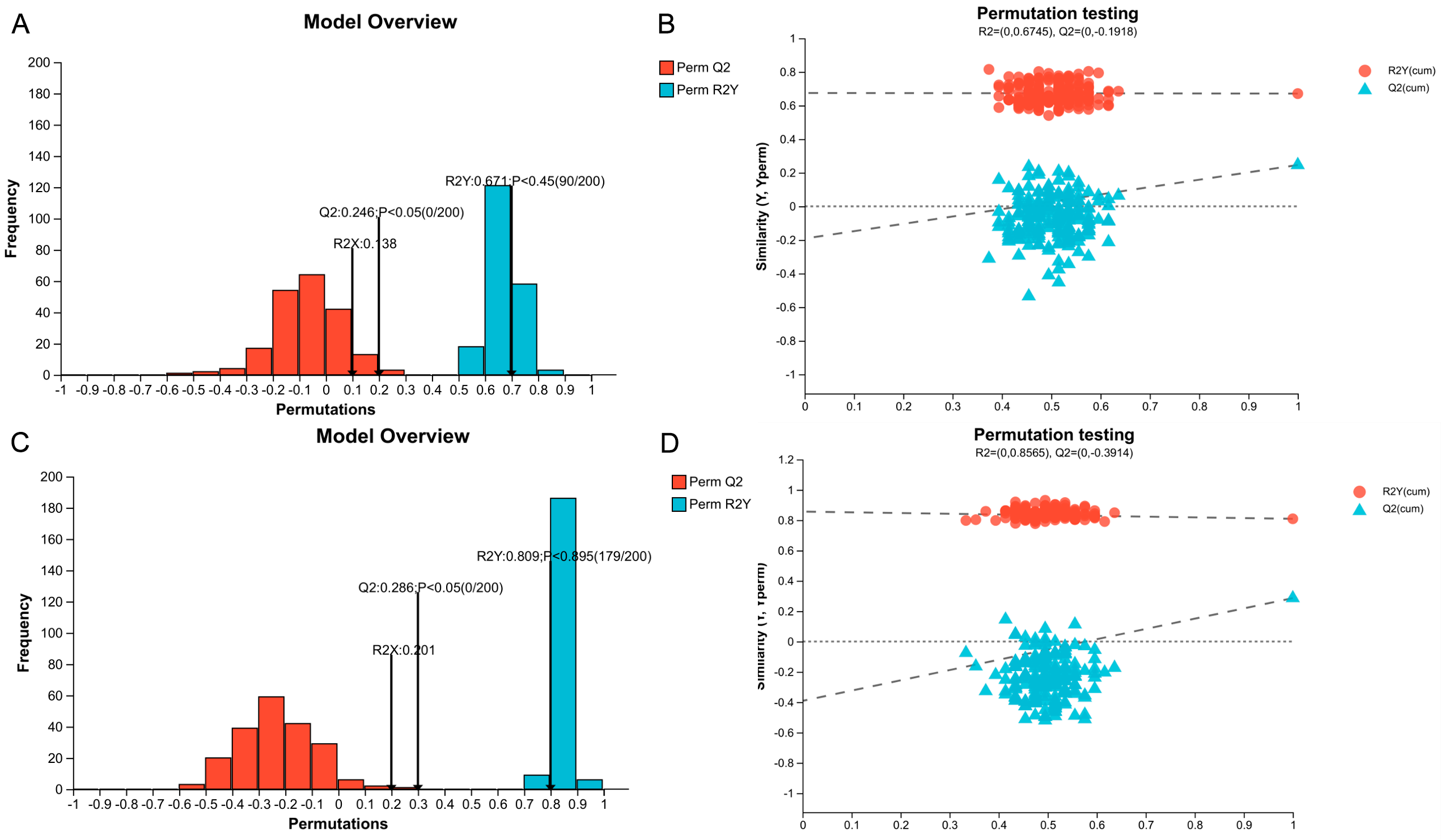

Supplement: Supplementary Figure 1 — Permutation test for PLS-DA and OPLS-DA. (A, B) Permutation test for PLS-DA. (C, D) Permutation test for OPLS-DA. PLS-DA, partial least squares discriminant analysis, OPLS-DA, orthogonal partial least squares discriminant analysis. [file Image1.jpeg]
