# Supplementary material for: Early prediction of gestational diabetes mellitus using machine learning-integrated metabolomic and clinical features
Source: Front Endocrinol (Lausanne). 2025 Nov 13;16:1687146. doi: 10.3389/fendo.2025.1687146 (PMC12658359; doi:10.3389/fendo.2025.1687146)
Supplement: Supplementary file 2 [file Table1.docx]

**Supplementary Table 1. Brief description of machine learning algorithms**

| **Model** | **Summary** |
| --- | --- |
| Decision tree | A decision tree is a supervised learning algorithm that constructs a hierarchical, tree-like model through the recursive partitioning of the dataset and is employed for tasks such as classification and regression^1^. It also serves as the essential models in more complicated models such as random forest and XGBoost^2,3^. In this model, the internal nodes correspond to feature evaluations, the branches denote possible outcomes of these evaluations, and the leaf nodes signify the predicted values. Critical components of decision tree construction include feature selection methods, such as information gain, and pruning techniques for optimization. The decision tree's primary advantages lie in its high interpretability and its applicability to both numerical and categorical data types^4^. |
| Random forest | Random forest is a powerful ensemble machine learning algorithm commonly employed for both classification and regression tasks. Based on the bagging technique, it constructs multiple decision trees by utilizing random subsets of both training data and features. The algorithm's final prediction is determined by combining the outputs from all individual decision trees through majority voting (for classification) or averaging (for regression)^2,5^. |
| XGBoost | XGBoost is an ensemble model of decision trees that is based on the concept of boosting. It involves constructing a series of decision tree models, with each tree being developed on the residuals of the preceding tree^3,6^. |
| Support vector machine | The support vector machine (SVM) is a sophisticated machine learning algorithm employed for various tasks, including classification, regression, and outlier detection. Its principal aim is to determine an optimal hyperplane that maximizes the margin between data points, thereby enhancing the model's predictive accuracy^7^. |
| Multilayer Perceptron | Multilayer Perceptron is a feedforward artificial neural network architecture, comprising an input layer, one or more hidden layers, and an output layer. Utilizing nonlinear activation functions in conjunction with the backpropagation algorithm, Multilayer Perceptron is capable of learning intricate data patterns, making them extensively applicable to tasks such as classification and regression^8,9^. |

**Reference**

1. Rosenblatt WH, Yanez ND. A Decision Tree Approach to Airway Management Pathways in the 2022 Difficult Airway Algorithm of the American Society of Anesthesiologists. *Anesth Analg*. 2022;134(5):910-915. doi:10.1213/ANE.0000000000005930

2. Breiman L. Random Forests. *Mach Learn*. 2001;45(1):5-32. doi:10.1023/A:1010933404324

3. Chen T, Guestrin C. XGBoost: A Scalable Tree Boosting System. In: *Proceedings of the 22nd ACM SIGKDD International Conference on Knowledge Discovery and Data Mining*. KDD ’16. Association for Computing Machinery; 2016:785-794. doi:10.1145/2939672.2939785

4. Jamshidi-Naeini Y, Brown AW, Mehta T, et al. A practical decision tree to support editorial adjudication of submitted parallel cluster randomized controlled trials. *Obes Silver Spring Md*. 2022;30(3):565-570. doi:10.1002/oby.23373

5. Yue S, Li S, Huang X, et al. Machine learning for the prediction of acute kidney injury in patients with sepsis. *J Transl Med*. 2022;20(1):215. doi:10.1186/s12967-022-03364-0

6. Li C, Liu M, Zhang Y, et al. Novel models by machine learning to predict prognosis of breast cancer brain metastases. *J Transl Med*. 2023;21:404. doi:10.1186/s12967-023-04277-2

7. Brereton RG, Lloyd GR. Support Vector Machines for classification and regression. *Analyst*. 2010;135(2):230-267. doi:10.1039/B918972F

8. Tolstikhin I, Houlsby N, Kolesnikov A, et al. MLP-Mixer: An all-MLP Architecture for Vision. Published online June 11, 2021. doi:10.48550/arXiv.2105.01601

9. Guo MH, Liu ZN, Mu TJ, Hu SM. Beyond Self-attention: External Attention using Two Linear Layers for Visual Tasks. Published online May 31, 2021. doi:10.48550/arXiv.2105.02358
