# Supplementary material for: Early prediction of gestational diabetes mellitus using machine learning-integrated metabolomic and clinical features
Source: Front Endocrinol (Lausanne). 2025 Nov 13;16:1687146. doi: 10.3389/fendo.2025.1687146 (PMC12658359; doi:10.3389/fendo.2025.1687146)
Supplement: Supplementary file 3 [file Table2.docx]

**Supplementary Table 2. All the demographic and clinical characteristics of participants**

| **Variables** | **Overall**  **(N=89)** | **GDM**  **(n=45)** | **NGT**  **(n=44)** | **P-value** |
| --- | --- | --- | --- | --- |
| Age (median [IQR]) | 32 [28, 35] | 32 [28, 35] | 32 [28, 34] | 0.479 |
| Height (mean (SD)) | 158.90 (5.07) | 159.07 (5.11) | 158.73 (5.09) | 0.754 |
| Weight (mean (SD)) | 58.89 (10.81) | 62.69 (11.38) | 55 (8.72) | 0.001 |
| Educational level (%) |  |  |  | 0.425 |
| Middle school | 15 (16.9) | 28 (62.2) | 30 (68.2) |  |
| High school | 15 (16.9) | 10 (22.2) | 5 (11.4) |  |
| Bachelor | 58 (65.2) | 7 (15.6) | 8 (18.2) |  |
| Postgraduate | 1 (1.1) | 0 (0.0) | 1 (2.3) |  |
| Occupation (%) |  |  |  | 0.965 |
| Employee | 28 (31.5) | 15 (33.3) | 13 (29.5) |  |
| Public | 22 (24.7) | 11 (24.4) | 11 (25.0) |  |
| Self-employed | 13 (14.6) | 7 (15.6) | 6 (13.6) |  |
| Service | 3 (3.4) | 1 (2.2) | 2 (4.5) |  |
| Unemployed | 23 (25.8) | 11 (24.4) | 12 (27.3) |  |
| Salary (%) |  |  |  | 0.387 |
| Less than 3K | 8 (9.0) | 11 (24.4) | 13 (29.5) |  |
| 3-5K | 24 (27.0) | 16 (35.6) | 20 (45.5) |  |
| 5-10K | 36 (40.4) | 12 (26.7) | 9 (20.5) |  |
| Greater than 10K | 21 (23.6) | 6 (13.3) | 2 (4.5) |  |
| Pregnancy planning (%) |  |  |  | 0.347 |
| Planed | 68 (76.4) | 32 (71.1) | 36 (81.8) |  |
| Unplaned | 21 (23.6) | 13 (28.9) | 8 (18.2) |  |
| Pre-pregnancy weight (mean (SD)) | 55.95 (9.39) | 59.16 (9.76) | 52.67 (7.82) | 0.001 |
| Pre-pregnancy PE (%) |  |  |  | 0.602 |
| Check | 50 (56.2) | 27 (60.0) | 23 (52.3) |  |
| Uncheck | 39 (43.8) | 18 (40.0) | 21 (47.7) |  |
| Methods.of.Conception (%) | |  |  |  |
| Assisted | 18 (20.2) | 8 (17.8) | 10 (22.7) |  |
| Nature | 71 (79.8) | 37 (82.2) | 34 (77.3) |  |
| Gravidity (median [IQR]) | 2 [1, 3] | 2 [2, 3] | 2 [1, 3] | 0.483 |
| Parity (median [IQR]) | 0 [0, 1] | 1 [0, 1] | 0 [0, 1] | 0.715 |
| Abortion (median [IQR]) | 0 [0, 1] | 0 [0, 1] | 0.50 [0, 1] | 0.975 |
| Adverse.Pregnancy.History (%) | |  |  |  |
| No | 49 (55.1) | 21 (46.7) | 28 (63.6) |  |
| Yes | 40 (44.9) | 24 (53.3) | 16 (36.4) |  |
| Natural.Childbirth (median [IQR]) | 0 [0, 1] | 0 [0, 1] | 0 [0, 1] | 0.208 |
| Caesarean.Section (median [IQR]) | 0 [0, 0] | 0 [0, 0] | 0 [0, 0] | 0.076 |
| Sleep.Duration (median [IQR]) | 8.0 [7.5, 9.0] | 8.0 [7.5, 9.0] | 8.0 [7.5, 8.5] | 0.445 |
| Nocturnal.Awakenings (median [IQR]) | 3 [1, 3] | 3 [1, 3] | 3 [3, 3] | 0.883 |
| Nighttime.Sleep (%) |  |  |  | 0.187 |
| After 1 | 5 (5.6) | 5 (11.1) | 0 (0.0) |  |
| After 12 | 8 (9.0) | 3 (6.7) | 5 (11.4) |  |
| Before 10 | 3 (3.4) | 1 (2.2) | 2 (4.5) |  |
| Before 11 | 30 (33.7) | 16 (35.6) | 14 (31.8) |  |
| Before 12 | 43 (48.3) | 20 (44.4) | 23 (52.3) |  |
| Nap (mean (SD)) | 1.0 [1.0, 2.0] | 1.0 [1.0, 2.0] | 1.0 [1.0, 2.0] | 0.713 |
| Physical.Labor (%) |  |  |  | 0.010 |
| Greater than 30min | 58 (65.2) | 23 (51.1) | 35 (79.5) |  |
| Less than 30min | 31 (34.8) | 22 (48.9) | 9 (20.5) |  |
| Stool (mean (SD)) | 1.0 [1.0, 2.0] | 1.0 [1.0, 2.0] | 1.0 [1.0, 2.0] | 0.989 |
| Menstrual.Irregularity (%) |  |  |  | 0.436 |
| Greater than 35d | 16 (18.0) | 10 (22.2) | 6 (13.6) |  |
| Less than 35d | 73 (82.0) | 35 (77.8) | 38 (86.4) |  |
| Hypertension (%) |  |  |  | 0.972 |
| No | 80 (89.9) | 41 (91.1) | 39 (88.6) |  |
| Yes | 9 (10.1) | 4 (8.9) | 5 (11.4) |  |
| Hyperlipidemia (%) |  |  |  | 0.979 |
| No | 84 (94.4) | 43 (95.6) | 41 (93.2) |  |
| Yes | 5 (5.6) | 2 (4.4) | 3 (6.8) |  |
| Hyperglycemia (%) |  |  |  | 0.079 |
| No | 76 (85.4) | 35 (77.8) | 41 (93.2) |  |
| Yes | 13 (14.6) | 10 (22.2) | 3 (6.8) |  |
| Hepatitis B virus infection (%) |  |  |  | 0.984 |
| No | 86 (96.6) | 44 (97.8) | 42 (95.5) |  |
| Yes | 3 (3.4) | 1 (2.2) | 2 (4.5) |  |
| Thalassemia (%) |  |  |  | 0.436 |
| No | 73 (82.0) | 35 (77.8) | 38 (86.4) |  |
| Yes | 16 (18.0) | 10 (22.2) | 6 (13.6) |  |
| Vitamin D taking (%) |  |  |  | 0.139 |
| No | 33 (37.1) | 15 (33.3) | 18 (40.9) |  |
| Vit D | 10 (11.2) | 8 (17.8) | 2 (4.5) |  |
| Vit D with multivitamin | 46 (51.7) | 22 (48.9) | 24 (54.5) |  |
| Polycystic ovary syndrome and insulin resistance (%) |  |  |  | 1.000 |
| No | 75 (84.3) | 38 (84.4) | 37 (84.1) |  |
| Yes | 14 (15.7) | 7 (15.6) | 7 (15.9) |  |
| Family history of hyperglycemia (%) |  |  |  | 0.687 |
| No | 63 (70.8) | 30 (66.7) | 33 (75.0) |  |
| Yes | 12 (13.5) | 7 (15.6) | 5 (11.4) |  |
| Parents or siblings | 14 (15.7) | 8 (17.8) | 6 (13.6) |  |
| Fasting plasma glucose (mean (SD)) | 4.58 (0.36) | 4.88 (0.20) | 4.27 (0.18) | <0.001 |
| Fins (median [IQR]) | 76.47 [51.15, 111.20] | 101.50 [67.32, 124.40] | 61.81 [42.85, 86.18] | <0.001 |
| Vitamin D level (median [IQR]) | 26.30 [21.00, 32.20] | 25.70 [20.90, 31.50] | 28.85 [21.38, 32.47] | 0.200 |
| White blood cell (mean (SD)) | 9.54 (2.02) | 9.48 (2.31) | 9.59 (1.70) | 0.785 |
| Neutrophils (mean (SD)) | 6.88 (1.72) | 6.80 (1.93) | 6.96 (1.50) | 0.662 |
| Lymphocytes (mean (SD)) | 2.15 (1.60) | 2.05 (0.60) | 2.26 (2.20) | 0.547 |
| Monocytes (mean (SD)) | 0.54 (0.16) | 0.54 (0.17) | 0.53 (0.16) | 0.771 |
| Eosinophils (mean (SD)) | 0.14 (0.10) | 0.13 (0.07) | 0.16 (0.13) | 0.258 |
| Basophils (mean (SD)) | 0.03 (0.02) | 0.02 (0.01) | 0.03 (0.02) | 0.258 |
| Red blood cell (mean (SD)) | 4.28 (0.51) | 4.33 (0.47) | 4.24 (0.56) | 0.442 |
| Hemoglobin (mean (SD)) | 121.15 (11.62) | 120.96 (11.69) | 121.34 (11.67) | 0.877 |
| Platelets (mean (SD)) | 269.94 (65.74) | 260.05 (75.93) | 280.05 (52.33) | 0.153 |
| Neutrophil to lymphocyte ratio (median [IQR]) | 13.06 [9.81, 16.46] | 12.18 [9.50, 15.94] | 13.57 [10.09, 16.60] | 0.506 |
| Alanine aminotransferase (mean (SD)) | 16.87 (12.75) | 14.83 (8.83) | 18.95 (15.63) | 0.128 |
| Blood urea nitrogen (mean (SD)) | 3.14 (3.12) | 3.63 (4.27) | 2.64 (0.86) | 0.132 |
| Uric acid (mean (SD)) | 240.31 (78.28) | 247.07 (89.06) | 233.41 (65.78) | 0.414 |
| Serum creatinine (mean (SD)) | 41.71 (7.27) | 41.11 (8.32) | 42.32 (6.05) | 0.437 |
| Estimated glomerular filtration rate (mean (SD)) | 169.42 (32.76) | 173.14 (38.29) | 165.62 (25.83) | 0.281 |
| Triglycerides (mean (SD)) | 1.94 (0.98) | 2.12 (0.95) | 1.67 (1.02) | <0.001 |
| Total cholesterol (mean (SD)) | 5.33 (1.20) | 5.28 (1.11) | 5.38 (1.29) | 0.699 |
| High-density lipoprotein cholesterol (median [IQR]) | 1.97 [1.77, 2.24] | 1.93 [1.74, 2.08] | 2.09 [1.84, 2.44] | 0.034 |
| Low-density lipoprotein cholesterol (median [IQR]) | 2.69 [2.29, 3.38] | 2.70 [2.20, 3.38] | 2.68 [2.30, 3.35] | 0.799 |
| Total body water (mean (SD)) | 27.92 (3.65) | 28.99 (3.86) | 26.82 (3.08) | 0.004 |
| Total intracellular water (mean (SD)) | 17.11 (2.28) | 17.80 (2.41) | 16.40 (1.93) | 0.003 |
| Total extracellular water (mean (SD)) | 10.81 (1.38) | 11.19 (1.47) | 10.42 (1.16) | 0.007 |
| Total body water of right arm (mean (SD)) | 1.80 (0.40) | 1.92 (0.43) | 1.67 (0.34) | 0.002 |
| Total body water of left arm (mean (SD)) | 90.78 (12.11) | 94.45 (12.99) | 87.03 (9.94) | 0.003 |
| Total body water of trunk (mean (SD)) | 1.74 (0.39) | 1.86 (0.40) | 1.62 (0.34) | 0.003 |
| Total body water of right leg (mean (SD)) | 87.82 (11.68) | 91.34 (12.21) | 84.22 (10.03) | 0.004 |
| Total body water of left leg (mean (SD)) | 16.91 (2.42) | 17.66 (2.52) | 16.14 (2.06) | 0.003 |
| Intracellular water of right arm (mean (SD)) | 95.13 (5.84) | 96.76 (6.20) | 93.45 (5.00) | 0.007 |
| Intracellular water of left arm (mean (SD)) | 5.75 (0.85) | 6.01 (0.89) | 5.49 (0.72) | 0.003 |
| Intracellular water of trunk (mean (SD)) | 92.72 (6.34) | 94.22 (6.70) | 91.19 (5.61) | 0.023 |
| Intracellular water of right leg (mean (SD)) | 5.74 (0.85) | 5.99 (0.89) | 5.48 (0.74) | 0.004 |
| Intracellular water of left leg (mean (SD)) | 92.45 (6.29) | 93.92 (6.46) | 90.95 (5.82) | 0.025 |
| Extracellular water of right arm (mean (SD)) | 1.40 (0.31) | 1.50 (0.33) | 1.30 (0.26) | 0.002 |
| Extracellular water of left arm (mean (SD)) | 1.36 (0.30) | 1.45 (0.31) | 1.26 (0.26) | 0.003 |
| Extracellular water of trunk (mean (SD)) | 13.20 (1.88) | 13.78 (1.96) | 12.60 (1.61) | 0.003 |
| Extracellular water of right leg (mean (SD)) | 4.49 (0.66) | 4.69 (0.69) | 4.29 (0.56) | 0.004 |
| Extracellular water of left leg (mean (SD)) | 4.48 (0.67) | 4.67 (0.70) | 4.28 (0.58) | 0.005 |
| Extracellular water to total body water ratio (mean (SD)) | 0.87 (0.20) | 0.93 (0.21) | 0.81 (0.16) | 0.002 |
| Extracellular water to total body water ratio of right arm (mean (SD)) | 0.84 (0.19) | 0.90 (0.19) | 0.78 (0.16) | 0.003 |
| Extracellular water to total body water ratio of left arm (mean (SD)) | 8.08 (1.17) | 8.46 (1.21) | 7.69 (1.01) | 0.002 |
| Extracellular water to total body water ratio of trunk (mean (SD)) | 2.75 (0.41) | 2.88 (0.43) | 2.62 (0.34) | 0.002 |
| Extracellular water to total body water ratio of right leg (mean (SD)) | 2.74 (0.41) | 2.86 (0.43) | 2.61 (0.35) | 0.003 |
| Protein (mean (SD)) | 7.39 (1.00) | 7.70 (1.07) | 7.08 (0.83) | 0.003 |
| Mineral content (mean (SD)) | 2.80 (0.37) | 2.89 (0.39) | 2.72 (0.33) | 0.026 |
| Body fat mass (mean (SD)) | 20.77 (6.65) | 23.11 (7.26) | 18.38 (4.99) | 0.001 |
| Skeletal lean mass (mean (SD)) | 35.77 (4.70) | 37.16 (4.98) | 34.34 (3.98) | 0.004 |
| Fat-free mass (mean (SD)) | 38.12 (5.00) | 39.58 (5.29) | 36.62 (4.23) | 0.005 |
| Skeletal muscle mass (mean (SD)) | 20.31 (2.98) | 21.22 (3.13) | 19.38 (2.51) | 0.003 |
| Body mass index (mean (SD)) | 23.29 (3.91) | 24.73 (4.05) | 21.81 (3.18) | <0.001 |
| Percentage of body fat (mean (SD)) | 34.48 (5.72) | 36.05 (6.30) | 32.87 (4.59) | 0.008 |
| InBody score (mean (SD)) | 68.43 (4.55) | 67.64 (5.56) | 69.23 (3.07) | 0.101 |
| Waist-hip ratio (mean (SD)) | 0.88 (0.05) | 0.89 (0.05) | 0.87 (0.04) | 0.034 |
| Visceral fat area (mean (SD)) | 103.74 (37.48) | 115.82 (41.60) | 91.39 (28.19) | 0.002 |
| Obesity degree (mean (SD)) | 110.91 (18.63) | 117.78 (19.27) | 103.89 (15.19) | <0.001 |
| Body cell mass (mean (SD)) | 24.50 (3.29) | 25.50 (3.48) | 23.48 (2.76) | 0.003 |
| Fat-Free mass index (mean (SD)) | 15.07 (1.68) | 15.61 (1.74) | 14.52 (1.45) | 0.002 |
| Fat mass index (mean (SD)) | 8.21 (2.55) | 9.12 (2.75) | 7.28 (1.94) | <0.001 |
| X50kHz right arm phase angle (mean (SD)) | 4.34 (0.50) | 4.48 (0.50) | 4.20 (0.47) | 0.008 |
| X50kHz left arm phase angle (mean (SD)) | 4.10 (0.51) | 4.24 (0.50) | 3.95 (0.48) | 0.006 |
| X50kHz trunk phase angle (median [IQR]) | 73.31 [73.10, 73.30] | 73.30 [73.10, 73.40] | 73.20 [73.12, 73.29] | 0.452 |
| X50kHz right leg phase angle (mean (SD)) | 4.90 (0.70) | 5.12 (0.68) | 4.67 (0.65) | 0.002 |
| X50kHz left leg phase angle (mean (SD)) | 4.77 (0.70) | 4.99 (0.68) | 4.53 (0.64) | 0.002 |
| X50kHz whole body phase angle (mean (SD)) | 4.61 (0.53) | 4.78 (0.52) | 4.44 (0.49) | 0.002 |
| Circumference of neck (cm, mean (SD)) | 32.31 (2.90) | 33.31 (2.98) | 31.28 (2.46) | 0.001 |
| Circumference of chest (cm, mean (SD)) | 87.05 (7.27) | 89.57 (7.43) | 84.47 (6.17) | 0.001 |
| Circumference of abdomen (cm, mean (SD)) | 81.78 (9.41) | 84.90 (9.97) | 78.60 (7.68) | 0.001 |
| Circumference of right hip (cm, mean (SD)) | 92.86 (6.38) | 95.26 (6.52) | 90.40 (5.24) | <0.001 |
| Circumference of right arm (cm, mean (SD)) | 28.76 (3.28) | 29.92 (3.31) | 27.56 (2.82) | 0.001 |
| Circumference of left arm (cm, mean (SD)) | 28.50 (3.25) | 29.66 (3.26) | 27.32 (2.82) | 0.001 |
| Circumference of right thigh (cm, mean (SD)) | 49.71 (4.23) | 51.34 (4.30) | 48.05 (3.47) | <0.001 |
| Circumference of left thigh (cm, mean (SD)) | 49.54 (4.19) | 51.16 (4.26) | 47.89 (3.44) | <0.001 |

Note: IQR, interquartile range; SD: standard deviation.
