# Supplementary material for: Early prediction of gestational diabetes mellitus using machine learning-integrated metabolomic and clinical features
Source: Front Endocrinol (Lausanne). 2025 Nov 13;16:1687146. doi: 10.3389/fendo.2025.1687146 (PMC12658359; doi:10.3389/fendo.2025.1687146)
Supplement: Supplementary file 4 [file Table3.docx]

**Supplementary Table 3. Features included in the machine learning model.**

| **Features** | **P-value** | **Score** | **Log2FC(GDM/NGT)** |
| --- | --- | --- | --- |
| [(1R,5R)-5-(6-Aminopurin-9-Yl)Cyclohex-3-En-1-Yl]Methanol | <0.001 | 0.215629914 | 0.0193 |
| N-Methyl-D-Aspartic Acid | <0.001 | 0.471566031 | 0.0499 |
| (2R,4R)-2-Phenylthiazolidine-4-Carboxylic Acid | <0.001 | 0.03217868 | 0.0677 |
| Lutein | <0.001 | 0.14217027 | 0.0500 |
| 4-Hydroxybutanoic Acid | <0.001 | 0.051892222 | 0.0287 |
| L-Gamma-Glutamyl-Beta-Phenyl-Beta-L-Alanine | <0.001 | 0.060008502 | 0.0194 |
| 6-Methoxymellein | <0.001 | 0.075508432 | -0.0137 |
| Prostaglandin B2 | <0.001 | 0.051153284 | -0.0788 |
| Glccer (D18:1/16:0) | <0.001 | 0.0283126 | -0.0495 |
| Dehydroacetic Acid | <0.001 | -0.111614768 | -0.2302 |
| 2,4-Dichlorophenylacetic Acid | <0.001 | -0.005488705 | 0.0387 |
| Fasting plasma insulin | <0.001 | -0.209946299 |  |
| Triglyceride | <0.001 | -0.123812688 |  |
| Intracellular water of left arm | <0.001 | 0.195376709 |  |

Note: FC: fold chage; GDM: gestational diabetes mellitus; NGT: normal glucose tolerance.
