# Supplementary material for: Early prediction of gestational diabetes mellitus using machine learning-integrated metabolomic and clinical features
Source: Front Endocrinol (Lausanne). 2025 Nov 13;16:1687146. doi: 10.3389/fendo.2025.1687146 (PMC12658359; doi:10.3389/fendo.2025.1687146)
Supplement: Supplementary file 5 [file Table4.docx]

**Supplementary Table 4. Best hyperparameter of the models.**

| **Models** | **Best hyperparameter** |
| --- | --- |
| Decision tree | criterion = “gini”  max_depth = 4  min_samples_leaf = 1  min_samples_split = 2 |
| Random forest | max_depth = 2  n_estimators = 60 |
| XGBoost | learning_rate = 0.12  max_depth = 1  n_estimators = 110  objective = “binary:logistic” |
| Support vector machine | C = 1.0  kernel = "linear" |
| Multilayer perceptron | activation = "logistic"  alpha = 0.01  hidden_layer_sizes = [100, 50]  learning_rate_init = 0.001  max_iter = 50  solver = "lbfgs" |
